# The Diagnosis of Plague

**Published:** 1900-10-13

**Authors:** 


### Hospital Clinics and Medical Progress.

### THE DIAGNOSIS OF PLAGUE.

Nothing could more forcibly show the truth of what we said last week as to the necessity of the sanitary authorities of this country being prepared to fight plague within their own boundaries, rather than trusting to its being kept out of the country by the efforts of port sanitary authorities, than the occurrence of a fatal case of the disease at Llandaff last Saturday, the patient having travelled across the country from a Tyne port, where he had arrived on September 23 on board a vessel from Rosario, River Plate.

In view then of the fact that anywhere in this much-travelled-over country a case of plague may crop up, and that it is of the extremest importance that any such "first case" should at once be isolated, all medical men should constantly bear plague in mind when considering the nature of any obscure febrile illness which they may meet with. The *Practitioner* for this month devotes a considerable amount of space to various subjects connected with plague, and from the remarks made by Dr. James Cantlie, Dr. David C. Rees, and Dr. Chalmers, we gather the following hints on the early diagnosis of the disease. The incubation period of plague may last as long as ten days, but from three to five days is the usual period which intervenes between exposure and the development of symptoms. Plague may be said to commence in one of three ways:—

1. A sudden rigor or chill may usher in the disease, accompanied or followed by high fever, headache, giddiness, thirst, vomiting, epigastric pain, extreme prostration, and aching in the back and limbs. The mental state may be one of apathy or of delirium.

2. In a certain number of cases no rigor occurs, but fever, headache, loss of appetite, and pains in the limbs obtain, with but little mental disturbance.

3. In children convulsions frequently usher in the attack, and may be the first indication that the child is ill.

Buboes may be present for the first, or their appearance may be delayed for a day or two; but, on the other hand, it must be remembered that none of the superficial groups of glands may be felt to be swollen during any period of the illness. It must also be noted that in its minor degrees plague may take the form of a very trifling malady, the constitutional symptoms being insignificant, the adenitis of a chronic character, and the infiltration of the surrounding tissues often absent. These ambulatory cases are at the root of all the trouble. They are apt to occur in considerable numbers at the beginning of an epidemic, and it is no doubt through them that the disease is spread about before it is discovered. A case of plague in its major form and in its early stage—the stage at which it is likely to be seen by the practitioner—may present the following symptoms:—

The patient may be found in a state of excitement, delirium, or partial apathy, and unable to answer questions put to him; the features are drawn and haggard, or they may appear expressionless, as if the patient were intoxicated. The eyes look sunken, the conjunctival vessels are infected, the face pale, and a dusky congestion prevails round the eyes, extending to the forehead and cheeks. The pulse will be found to beat at the rate of 100 or more, and to be full and not readily compressed; but it soon becomes rapid, and small or markedly dicrotic. The breathing is hastened from the beginning of the illness, and not infrequently hiccough or a short, dry cough is noticeable. The temperature is generally raised, but there is nothing distinctive about it. It may be normal or subnormal, but in these apyretic cases no doubt there has been a temporary initial rise.

The tongue appears swollen, and the dorsum is coated with a general white fur, leaving the sides and tip red and uncovered; sore throat may be complained of, one or both tonsils may be enlarged, and vomiting and nausea are often distressing. The abdomen is frequently tender and tense. Constipation is the rule, but diarrhœa is not unknown. The urine is unaltered at the onset, but may be scanty. The patient may be found lying flat on the back, or if a bubo is causing pain he may be sitting up in bed with the knees almost at the chin, or he may lie on one side with the lower limb on which the buboes exist in the position of flexion. The glands in the groin, axilla, or neck may be found swollen and tender, or tenderness alone may exist, elicited only when deep pressure is made in the region of the glands.

Thus there is nothing specific about these early symptoms unless buboes are present. But buboes are seldom an initial sign. Still, the formation of buboes is the principal clinical evidence that a patient is suffering from plague, and they occur in three-fourths of the patients. Perhaps they always occur somewhere or another, although not found. A bubo may be apparent in the groin, in the armpit, or in the neck from the very commencement of the illness, although proof of its presence may only be elicited by careful examination, or a mere tenderness over a particular gland may be all that is to be found. When buboes are present before the patient becomes indifferent to suffering they cause pain of a sharp, lancinating character. In the majority of cases the buboes become evident during the first thirty-six hours, but it may be the third day or later before they are discoverable. In 50 per cent. of the cases showing them they are in the groin, in 30 per cent. in the axilla, in 15 per cent. in the neck, and occasionally in the elbow and popliteal space. Unilateral adenitis is the rule, and simultaneous enlargement of glands in different parts of the body is not a common occurrence. The size of the glandular swelling is obscured by œdema, or hæmorrhage, or sero-sanguinolent effusion extending for a considerable distance around. The size of the swelling may be that of a bean or walnut only, or in outline and dimension "it may resemble a thick bun." In searching for enlarged glands attention should first be directed to the vertical set of inguinal glands, just below Poupart's ligament, deeply placed below the fascia lata. As adenitis proceeds the tissues around become matted together, œdematous and discoloured for a considerable distance, and in no long time the skin reddens, then sloughs, and a large cavity is left which discharges pus freely. The pain attending the adenitis may be excruciating, but as the patient's mental state becomes affected evidence of the gland being painful can only be elicited by pressure over it. The attitude assumed by the patient, however, often shows where there is pain or tension.

Take it altogether, it may be said that where plague occurs in a locality in which it is unlooked for, it is a disease difficult of recognition in its early stages. Two or three persons may die of pneumonia running a rapid course, within a few days of each other, without suggesting the idea of plague unless that disease is in one's mind. A woman in the pregnant state may abort and die (the usual sequence in plague), a child may be suddenly convulsed, glandular fever may occur amongst children, or a patient may develop abdominal symptoms of a typhoid character, without the fact of plague being the cause occurring to anyone. But when plague exists in a locality the diagnosis of a typical case is easy. The pneumonic type of the disease is, however, difficult of diagnosis, especially if the thought of plague is not present in one's mind. Assistance in suspecting plague in such cases is afforded by the rapid onset of the lung lesion without any previous bronchial symptoms, by the sudden attack of prostration, fever, and general symptoms, and by inspection of the sputum, which will be found watery and profuse, and tinged by or mixed with blood. It is only by microscopic examination of the sputum, however, that the presence of the disease can be actually diagnosed. In fact, the diagnosis of no case of plague is complete unless the bacillus pestis is proved to exist. In the pneumonic form the diagnosis may readily be made by microscopic examination of cover-glass preparations prepared from the sputum. In the bubonic form all that one has to do to obtain a specimen is to make a small incision into the gland, the skin having been previously rendered aseptic. Anæsthesia may be obtained by freezing, but no chemical antiseptics should be employed in the operation, except for the preliminary cleansing of the skin. A small piece of the gland tissue should be placed at once into a sterilised test-tube. Broth tubes may also be inoculated, and cover-glass preparations made if these are at hand, and the whole should at once be sent to a laboratory for examination, the case in the meantime being isolated. If incision of the glands is not thought desirable, blood films may be obtained by hypodermic puncture of the buboes. The needle used should afterwards be at once destroyed by fire. To put the whole thing in a nutshell, what all medical men have to look out for just now is fever with glandular swellings. In the last report drawn up by the late Sir Richard Thorne, he said: "Fever with glandular swellings prevailed in Bombay before it was recognised that plague had reached that city; and it is impossible to read the medical history of this disease in almost every part of the world, without being impressed with the frequency with which recognised plague has been preceded by ailments of such slight severity, involving some bubonic enlargement of glands, and some rise in body temperature, as to mask the real nature of the malady." Fever, then, of whatever type, complicated with glandular swelling of whatever intensity, is what medical practitioners all over the country have to look out for: in addition to which all ill-defined febrile attacks should be carefully investigated.

